# Prognostic role of carcinoembryonic antigen and carbohydrate antigen 19-9 in metastatic colorectal cancer: a *BRAF*-mutant subset with high CA 19-9 level and poor outcome

**DOI:** 10.1038/s41416-018-0115-9

**Published:** 2018-06-06

**Authors:** Maria Thomsen, Eva Skovlund, Halfdan Sorbye, Nils Bolstad, Kjell Johannes Nustad, Bengt Glimelius, Per Pfeiffer, Elin H. Kure, Julia S. Johansen, Kjell Magne Tveit, Thoralf Christoffersen, Tormod Kyrre Guren

**Affiliations:** 1 Department of Oncology, Oslo University Hospital, Oslo, Norway; 2 Institute of Clinical Medicine, Faculty of Medicine, University of Oslo, Oslo, Norway; 3 Department of Public Health and Nursing, Norwegian University of Science and Technology, Trondheim, Norway; 4 Department of Oncology, Haukeland University Hospital and Department of Clinical Science, University of Bergen, Bergen, Norway; 5 Department of Medical Biochemistry, Oslo University Hospital, Oslo, Norway; 6 Department of Immunology, Genetics and Pathology, Uppsala University, Uppsala, Sweden; 7 Department of Oncology, Odense University Hospital, Odense, Denmark; 8 Institute of Clinical Research, University of Southern Denmark, Odense, Denmark; 9 Department of Cancer Genetics, Institute for Cancer Research, Oslo University Hospital, Oslo, Norway; 10 Departments of Oncology and Medicine, Herlev and Gentofte Hospital, Copenhagen University Hospital, Copenhagen, Denmark; 11 K.G.Jebsen Colorectal Cancer Research Centre, Oslo University Hospital, Oslo, Norway; 12 Department of Pharmacology, Institute of Clinical Medicine, Faculty of Medicine, University of Oslo, Oslo, Norway

**Keywords:** Colon cancer, Rectal cancer, Prognostic markers, Colorectal cancer

## Abstract

**Background:**

Mutation status of *RAS* and *BRAF*, as well as serum levels of carcinoembryonic antigen (CEA) and carbohydrate antigen 19-9 (CA 19-9), are biomarkers used in clinical management of patients with gastrointestinal cancers. This study aimed to examine the prognostic role of these biomarkers in a patient population that started first-line chemotherapy for unresectable metastatic colorectal cancer (mCRC) in the NORDIC-VII study.

**Methods:**

CEA and CA 19-9 were measured in serum samples from 545 patients obtained before the start of chemotherapy. Four hundred and ninety-four patients had detectable levels of carbohydrate antigen 19-9 (CA 19-9). *RAS* (exons 2–4) and *BRAF* (V600E) mutation status were available from 440 patients. Overall survival (OS) was estimated in patient groups defined by serum CEA or CA 19-9 levels using cut-off values of 5 µg/L and 35 kU/L, respectively, in the total population and in subgroups according to *RAS* and *BRAF* mutation status.

**Results:**

For both CEA and CA 19-9, elevated serum levels were associated with reduced OS in adjusted analyses which included *RAS* and *BRAF* mutation status, baseline World Health Organization performance status, and levels of alkaline phosphatase and C-reactive protein. The negative prognostic information provided by an elevated CA 19-9 level was particularly marked in patients with *BRAF* mutation (hazard ratio = 4.35, interaction *P* = 0.003, in an adjusted model for OS).

**Conclusions:**

High baseline serum concentrations of CEA and CA 19-9 provide independent information of impaired prognosis in mCRC. In patients with *BRAF*-mutant tumours, elevated serum CA 19-9 may identify a subgroup with highly aggressive disease and could contribute to improving therapeutic decisions.

## Introduction

Colorectal cancer (CRC) is the third most common cancer and 40–50% of patients with CRC will develop distant metastases.^1^ Systemic therapy is the main treatment option for metastatic CRC (mCRC), and better treatment during the past decades has resulted in improved survival. However, mCRC is a heterogeneous disease, and selecting patients for optimal treatment is a challenge. Many factors determine the outcome. Development of robust and easily available biomarkers may help to individualise treatment.

Mutations in *RAS* (exons 2–4) and particularly *BRAF* (V600E) in the tumour cells are associated with impaired prognosis,^2–6^ and mutation status is routinely used in the clinical management of patients with mCRC. About 50% of the patients have *RAS* mutations which predict lack of effect from systemic treatment with epidermal growth factor receptor (EGFR) antibodies.^7,8^ The activating *BRAF* (V600E) mutation is found in 5–20% of the tumours in mCRC patient cohorts.^3,5,6,9^

Assays of certain glycoproteins and carbohydrates expressed by cancer cells are used in the clinical management of patients with gastrointestinal malignancies.^10,11^ Carcinoembryonic antigen (CEA), a glycoprotein belonging to a group of adhesion molecules, is produced in the epithelium of the large intestine and may be involved in malignancy.^12^ CEA has an established role as a biomarker in diagnosis, treatment and surveillance in CRC,^10^ and elevated serum levels of CEA are associated with inferior prognosis.^10,13,14^ Carbohydrate antigen 19-9 (CA 19-9), a tetrasaccharide carbohydrate also termed sialyl Lewis a, synthesised by gastrointestinal epithelium, is an established serum biomarker for monitoring treatment of patients with pancreatic cancer. While there is some evidence of a relationship between elevated CA 19-9 levels and outcome in CRC,^15–17^ its role in the management of patients with mCRC is so far unclear.^10,18^ Little is also known about the prognostic information that can be obtained from the serum levels of these two biomarkers when analysed in relation to tumour *RAS* and *BRAF* mutation status.

One particular focus of the present study is the group of patients with *BRAF*-mutant tumours. Although the presence of *BRAF* mutation in general is known to be associated with an inferior outcome of mCRC,^2,4,6^ there is a significant heterogeneity within the *BRAF*-mutant population.^9,19,20^ It is important to learn more about the basis for this heterogeneity, since it is conceivable that optimal therapeutic care might differ for these patients.^20,21^ We have previously shown that in mCRC patients with *BRAF* mutations, evidence of a systemic inflammation predicted an extremely aggressive disease with very short survival.^22^ Furthermore, during treatment of mCRC patients, we have accidentally observed high serum levels of CA 19-9 in several patients with *BRAF*-mutant tumours. The aim of the present study was to explore the prognostic information of serum CEA and CA 19-9 in patients with mCRC, especially as related to *RAS* and *BRAF* mutation status, and with emphasis on CA 19-9 in patients with *BRAF*-mutant tumours.

## Materials and methods

### Patients

The randomised NORDIC-VII study investigated the effects of combining cetuximab with the Nordic FLOX regimen with bolus 5-flououracil/folinic acid and oxaliplatin^23^ in previously untreated patients with unresectable mCRC.^24^ A total of 566 patients were randomly assigned to receive FLOX, cetuximab plus FLOX or cetuximab combined with intermittent FLOX as first-line palliative chemotherapy. There were no statistically significant differences in outcome (progression-free survival (PFS), overall survival (OS), overall response rate or secondary R0 resection of metastases) between the different treatment arms in the NORDIC-VII study.^6,24^ In the present study, data were analysed across the three treatment arms.

**Fig. 1:**
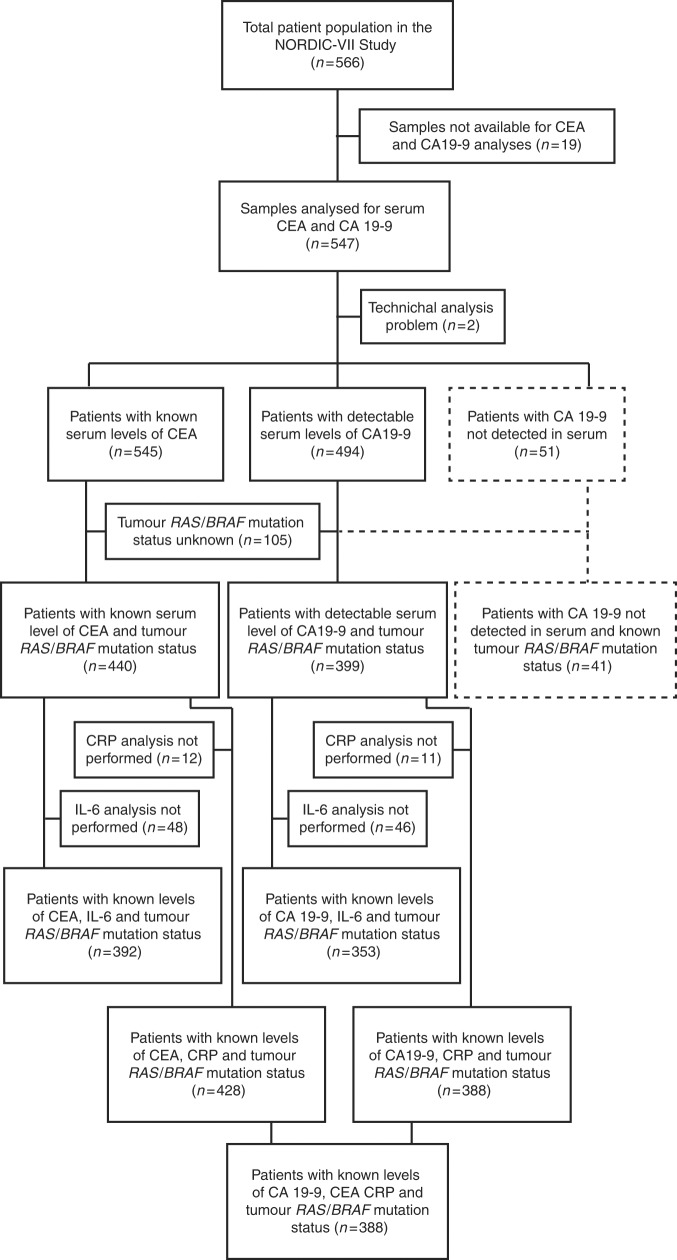
CONSORT diagram. Study populations

The CONSORT diagram in Fig. 1 describes the different patient populations. CA 19-9 and CEA were analysed in serum from 545 patients. Tumour *RAS*/*BRAF* mutation status was known in 440 of these patients, and further information about plasma C-reactive protein (CRP) and interleukin-6 (IL-6) level was available in 428 and 392 patients, respectively. The different prognostic clinicopathological markers were defined as alkaline phosphatase (ALP) level normal or elevated (above upper normal limit (UNL) based on institutional reference values at the study sites), CRP (below or above 10 mg/L), platelet count (below or above 400/nL) and white blood cell count (below or above 10/nL). These cut-off values have been used in large mCRC phase III trials and in prognostic indexes.^3,8,25,26^ The baseline demographics and clinical characteristics of the patients are shown in Table 1.

**Table 1:** Baseline patient characteristics

Variables	Study populations					
	Total (*n* = 566)	CEA/CA 19-9 analysed (*n* = 545)	CA 19-9 detected (*n* = 494)	CA 19-9 not detected (*n* = 51)	CEA analysed *RAS*/*BRAF* analysed (*n*= 440)	CA 19-9 detected *RAS*/*BRAF* analysed (*n* = 399)
Age (years)						
Median (min, max)	62 (24, 75)	62 (24, 75)	62 (24, 75)	60 (30, 75)	62 (24, 75)	62 (24, 75)
Gender, *n* (%)						
Male	334 (59)	322 (59)	292 (59)	30 (59)	265 (60)	241 (60)
Female	232 (41)	223 (41)	202 (41)	21 (41)	175 (40)	158 (40)
WHO performance status, *n* (%)						
0	380 (67)	367 (67)	328 (66)	39 (76)	295 (67)	263 (67)
1	162 (29)	155 (28)	145 (29)	10 (20)	126 (29)	119 (29)
2	24 (4)	23 (4)	21 (4)	2 (4)	19 (4)	17 (4)
Location primary tumour, *n* (%)						
Colon	333 (59)	321 (59)	297 (60)	24 (47)	265 (60)	244 (61)
Rectum	233 (41)	224 (41)	197 (40)	27 (53)	175 (40)	155 (39)
Previous surgery, *n* (%)						
Primary tumour resected	382 (67)	369 (68)	334 (68)	35 (69)	335 (76)	303 (76)
Intact primary tumour	184 (33)	176 (32)	160 (32)	16 (31)	105 (24)	96 (24)
Prior pelvic RT, *n* (%)						
Yes	80 (14)	75 (14)	68 (14)	7 (14)	66 (15)	60 (15)
No	486 (86)	470 (86)	426 (86)	44 (86)	374 (85)	339 (85)
Prior adjuvant CT, *n* (%)						
Yes	51 (9)	49 (9)	42 (9)	7 (14)	44 (10)	38 (10)
No	515 (91)	496 (91)	452 (91)	44 (86)	396 (90)	351 (90)
Time of metastases, *n* (%)						
Synchronous	402 (71)	389 (71)	347 (70)	42 (82)	298 (68)	266 (67)
Metachronous	164 (29)	156 (29)	147 (30)	9 (18)	142 (32)	133 (33)
Number of metastatic sites, *n* (%)						
1 site	162 (29)	155 (28)	137 (28)	18 (35)	130 (30)	115 (29)
>1 sites	404 (71)	390 (72)	357 (72)	33 (65)	310 (70)	284 (71)
Alkaline phophatase level, *n* (%)						
Normal	298 (53)	286 (52)	258 (48)	26 (55)	240 (55)	217 (54)
>UNL	268 (47)	259 (48)	236 (48)	23 (45)	200 (45)	182 (46)
Platelet count, *n* (%)						
≤400/nL	398 (70)	383 (70)	347 (70)	36 (71)	315 (72)	287 (72)
>400/nL	168 (30)	162 (30)	147 (30)	15 (29)	125 (28)	112 (28)
White blood cell count, *n* (%)						
≤10/nL	428 (76)	416 (76)	369 (75)	44 (86)	345 (78)	309 (77)
>10/nL	138 (24)	132 (24)	125 (25)	7 (14)	95 (22)	90 (23)
RAS/BRAF mutation status, *n* (%)						
* RAS*/*BRAF* wild-type				15^a^ (37)	186 (42)	171 (43)
* RAS* mutation				22^a^ (54)	201 (46)	179 (45)
* BRA*F mutation				4^a^ (10)	53 (12)	49 (12)
CRP level, *n* (%)						
≤10 mg/L				18^b^ (45)	194^d^ (45)	176^f^ (45)
>10 mg/L				22^b^ (55)	234^d^ (55)	212^f^ (55)
IL-6 level, *n* (%)						
<5.6 ng/L				21^c^ (54)	193^e^ (49)	172^g^ (49)
≥5.6 ng/L				18^c^ (46)	199^e^ (51)	181^g^ (51)

*CRP* C-reactive protein, *CEA* carcinoembryonic antigen, *CA* carbohydrate antigen, *CT* chemotherapy, IL-6 interleukin-6, *PS* performance status, *RT* radiation therapy, *UNL* upper normal limit, *WHO* World Health Organization

^a^Analysed for *RAS*/*BRAF* mutations (*n* = 41).

^b^CRP (*n* = 40).

^c^IL-6 (*n* = 39).

^d^CRP (*n* = 428).

^e^IL-6 (*n* = 392).

^f^CRP (*n* = 388).

^g^IL-6 (*n* = 353).

### Mutation analyses of *KRAS*, *NRAS* and *BRAF*

Genomic DNA was extracted from formalin-fixed paraffin-embedded 10 µm tumour tissue sections (65–70% (median) tumour cells) using QIAamp DNA Micro Kit (Cat. 56304, Qiagen). Tumour DNA was screened for the presence of *RAS* (exons 2–4) and *BRAF* (V600E) mutations as previously described.^6,24^

### Measurement of CEA and CA 19-9

Fresh-frozen serum samples were obtained from patients before the start of treatment and after 8 weeks of treatment. The samples were thawed and mixed before analysis of CEA (Ref. No. 04491777 190) and CA 19-9 (Ref. No. 11776193 122) on the Cobas e601 instrument (Roche Diagnostics GmbH, Mannheim, Germany). Established Norwegian reference values were used, with cut-off values 5 µg/L for CEA and 35 kU/L for CA 19-9. Patients with undetectable levels of CA 19-9 (i.e., below the lower analytical detection limit; 5 kU/L) were classified as a separate category in the primary statistical analyses. The results are presented in accordance with the REMARK guidelines.^27^

### Statistical analyses

The statistical analyses were performed using IBM SPSS (version 23, IBM Corp., Armonk, NY, USA). Demographic data were described with median and range (continuous variables) or with proportions and percentages (categorical variables). The prognostic values of different serum levels of CEA and CA 19-9 and survival were assessed by Kaplan–Meier method, log-rank test and Cox proportional hazards model. Separate analyses of the effect of World Health Organization (WHO) performance status, ALP level, number of metastatic sites, CRP level and tumour *RAS*/*BRAF* mutation status were performed. Only variables statistically significant (*P* < 0.05) in these analyses were included in the multivariable analyses, and models were restricted to include statistically significant variables only. An interaction term was included in the Cox model to explore the effect of mutation status on the prognostic effect of serum levels of CEA and CA 19-9.

## Results

### Clinical characteristics and serum levels of CEA and CA 19-9

**Fig. 2:**
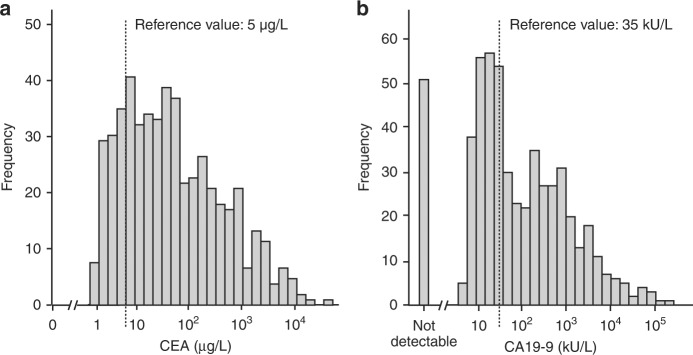
Histograms. Distribution of serum level of (**a**) CEA (median: 36 µg/L; range: 0.5–32,916 µg/L) and (**b**) CA 19-9 (median 50 kU/L; range 5–185,991 kU/L). Fifty-one patients had undetectable levels of CA 19-9 in the serum

The frequency distribution of the levels of CEA and CA 19-9 is shown in Fig. 2a, b, respectively. From 545 analysed patients, 96 (18%) had serum levels of CEA below 5 µg/L and 449 (82%) had elevated CEA levels (≥5 µg/L). Furthermore, 494 patients had detectable serum levels of CA 19-9 in their serum samples. Of these patients, 188 (38%) had a serum level of CA 19-9 below 35 kU/L and 306 (62%) had elevated CA 19-9 (≥35 kU/L). From 51 patients (9%), CA 19-9 was below the detection limit.

For both CEA and CA 19-9, elevated serum levels were associated with impaired baseline clinical patient characteristics, such as inferior WHO performance status, intact primary tumour, synchronous metastases, elevated ALP level, elevated platelet (>400/nL) and white blood cell (>10/nL) counts and elevated CRP (>10 mg/L) (Supplementary Table 1). Elevated CA 19-9 was somewhat more frequent in patients with colon cancer as compared to rectal cancer, while there was no association between CEA level and origin of primary tumour. Furthermore, 33% and 71% of the patients with lung-only metastases had levels of CEA below 5 µg/L and CA 19-9 below 35 kU/L, respectively. No relationship between other metastatic sites and serum levels of CEA or CA 19-9 was observed.

In patients with elevated serum CA 19-9 (≥35 kU/L), the median was numerically higher in those with with *BRAF*-mutant tumours (853 kU/L) compared to those with *RAS*-mutant or *RAS/BRAF* wild-type tumours (336 and 254 kU/L, respectively). Furthermore, 8 of the 16 patients with an isolated elevated CA 19-9 (i.e., CEA not increased) had *BRAF*-mutant tumours.There was no difference in median CEA value between subgroups based on *RAS* or *BRAF* mutation status.

### Serum level of CEA as an independent prognostic biomarker

**Fig. 3:**
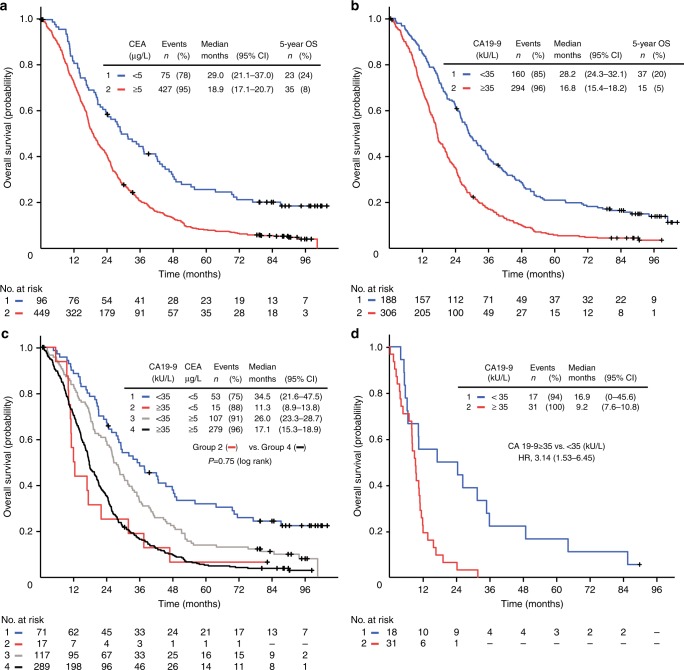
Overall survival. OS in 545 and 494 patients with different baseline serum levels of CEA and CA 19-9, respectively (**a**–**c**). **a** Serum level of CEA <5 µg/L or CEA ≥5 µg/L. **b** Serum level of CA 19-9 <35 kU/L or CA 19-9 ≥35 kU/L. **c** Serum level of CA 19-9 level <35 kU/L or CA 19-9 ≥35 kU/L in patients with CEA level <5 µg/L or CEA ≥5 µg/L. **d** OS in 51 patients with *BRAF*-mutant tumours; CA 19-9 level <35 kU/L or CA 19-9 ≥35 kU/L. *OS* overall survival, *HR* hazard ratio, *C*I confidence interval

As expected, there was a relationship between increasing serum levels of CEA (grouped in quartiles) and impaired survival (log-rank test for trend, *P* < 0.001). Patients with elevated CEA (≥5 µg/L) and low CEA (<5 µg/L) had a median OS of 19 and 29 months, respectively (hazard ratio (HR), 1.85 (95% confidence interval (CI) 1.45–2.38); *P* < 0.001). Furthermore, 24% of the patients with serum CEA below 5 µg/L survived at least 5 years compared to 8% of those with an elevated CEA level (Fig. 3a), although there was no difference in frequency of secondary metastasectomy in the two subgroups (9% and 8%, respectively). The prognostic information of baseline CEA was further confirmed in an adjusted model including other prognostic markers and clinical characteristics believed to be potential confounders, including WHO performance status, ALP and CRP levels and *RAS/BRAF* mutation status (adjusted HR, 1.78 (95% CI 1.34–2.37); *P* < 0.001), as shown in Supplementary Table 2.

### Serum CA 19-9 level as a prognostic biomarker

There was a statistically significant relationship between levels of CA 19-9 (grouped in quartiles) and OS (log-rank test for trend, *P* < 0.001). Figure 3b shows that an elevated serum CA 19-9 (≥35 kU/L) at baseline was associated with impaired outcome, with median OS 17 months, compared to patients with CA 19-9 below 35 kU/L who had a median OS of 28 months (HR = 1.95 (95% CI 1.60–2.38); *P* *<* 0.001). Thirty-seven (20%) of the patients with serum CA 19-9 level below 35 kU/L survived beyond 5 years, compared to 5% of the patients with elevated CA 19-9, with no difference in resection rates of metastases in the two groups (10% and 8%, respectively). This association between elevated CA 19-9 and impaired OS was found both in patients with low and elevated serum level of CEA (Fig. 3c). Essentially, a similar association was demonstrated in the groups of patients with known *RAS*/*BRAF* mutation status or *RAS*/*BRAF* mutation and plasma CRP level (not shown). There was a trend that the prognostic value of elevated CA 19-9 was more pronounced in patients with colon cancer compared to rectal cancer, but no statistically significant interaction between CA 19-9 and site on OS was detected (not shown).

The baseline serum level of CA 19-9 or CEA did not influence the initial chemotherapy response, measured as rates of overall response, disease control, direct progression or secondary resection of metastases, neither in the total patient population nor in subgroups defined by *RAS* or *BRAF* mutations (Supplementary Tables 3 and 4).

Paired serum samples obtained before the start of treatment and after four cycles of chemotherapy (approximately 8 weeks of treatment) were available from 384 and 260 patients with elevated baseline levels of CEA and CA 19-9, respectively. There was an association between treatment response and a drop in serum levels for both markers (Supplementary Table 5). However, the change, absolute or relative, in serum levels of CEA and CA 19-9 did not predict prognosis better than the baseline level before the start of treatment (not shown).

For 51 patients (9%), CA 19-9 was not detectable in the serum samples as judged by the lower reference value. However, in this subgroup 33 of the patients (6% of the analysed population) had no analytic signal or very low values classified as analytic noise, whereas 18 patients had CA 19-9 values between 3 and 5 kU/L, below the lower analytical reference value. These data suggest that at least 2/3 of the patients with no detectable CA 19-9 had Le (a-b-) genotype in the Lewis blood group system, consistent with the reported frequency of 5% to 10% of individuals lacking expression of Lewis antigens in Caucasian populations.^18,28^ The subgroup of patients for whom CA 19-9 could not be detected showed clinical features that were essentially similar to the total population (Table 1) and there was no statistically significant difference between the subcategories and clinical outcome (Supplementary Figure 1A). Sensitivity analyses including these patients with CA 19-9 assigned the value 0 did not significantly alter the results of the study (not shown).

### Serum levels of CA 19-9 and tumour *BRAF* mutation status

The data showed that the prognostic information from elevated levels of CA 19-9 differed depending on the *RAS* and *BRAF* mutation status of the tumour, with a statistically significant interaction in adjusted analyses (*P* = 0.003). In contrast, there was no statistically significant interaction between serum CEA level and *RAS*/*BRAF* mutation status (*P* = 0.27).

Elevated levels of CA 19-9 were associated with a particularly impaired OS in *BRAF*-mutant cancers. The patients with *BRAF*-mutant tumours had an estimated median OS of 17 and 9 months in unadjusted analyses stratified on low and elevated serum levels of CA 19-9, respectively, and the two groups differed significantly in the tail of the Kaplan–Meier curves (Fig. 3d). In an adjusted proportional hazards model including WHO performance status and levels of ALP, CEA and CRP, an elevated level of serum CA 19-9 was associated with a significant relative reduction in survival (adjusted HR = 4.35 (95% CI 2.89–8.28) in patients with tumours harbouring *BRAF* mutation (Table 2).

**Table 2:** Association between prognostic factors and overall survival in 388 patients with detectable serum levels of CA 19-9^a^

Variables	HR	95% CI	*P* value
*RAS*/*BRAF* mutation status			
* RAS*/*BRAF* wild-type			0.003^b^
CA 19-9 <35 kU/L	1		
CA 19-9 ≥35 kU/L	1.35	0.96–1.91	
* RAS* mutation			
CA 19-9 <35 kU/L	1		
CA 19-9 ≥35 kU/L	1.43	1.00–2.05	
* BRAF* mutation			
CA 19-9 <35 kU/L	1		
CA 19-9 ≥35 kU/L	4.35	2.89–8.28	
CEA level			
<5 µg/L	1		0.036
≥5 µg/L	1.41	1.02–1.94	
WHO performance status			
0	1		0.004
1	1.37	1.07–1.76	
2	2.56	1.31–3.89	
Alkaline phosphatase level			
Normal	1		0.003
>UNL	1.44	1.13–1.82	
CRP level			
Per category^c^	1.17	1.05–1.31	0.006

*CI* confidence interval, *CEA* carcinoembryonic antigen, *CA* carbohydrate antigen, *CRP* C-reactive protein, *HR* hazard ratio, *OS* overall survival, *UNL* upper normal limit, *WHO* World Health Organization

^a^ Adjusted model including the interaction between tumour *RAS/BRAF* mutation status and CA 19-9.

^b^ Interaction *P*.

^c^ CRP categories: from 0 to 10 mg/L, from 10 to 30 mg/L, from 30 to 60 mg/L, from 60 mg/L and higher.

In the NORDIC-VII patient cohort, we previously reported that an elevated level of IL-6 was associated with an impaired prognosis, especially in patients with *BRAF*-mutant tumours.^22^ To examine the relationship between CA 19-9 and IL-6, we included information about IL-6 levels (cut-off value 5.6 ng/L) in the analysis of how CA 19-9 predicts OS. Table 3, showing results from patients with *BRAF*-mutant tumours, demonstrates that at high levels of IL-6 with poor survival there was still a further impaired outcome associated with high CA 19-9. This suggests that IL-6 and CA 19-9, at least partly, reflect independent mechanisms associated with inferior prognosis.

**Table 3:** Outcome in 42 patients with *BRAF*-mutant tumours: OS in subgroups defined by baseline level of CA 19-9 and IL-6

Variables	*n*	Events	Median OS (95% CI)	HR (95% CI)
CA 19-9 <35 kU/L IL-6 <5.6 ng/L	10	9	26.0 (15.2–36.8)	1
CA 19-9 ≥35 kU/L IL-6 <5.6 ng/L	7	7	11.8 (9.4–14.1)	4.3 (1.3–14.0)
CA 19-9 <35 kU/L IL-6 ≥5.6 ng/L	6	6	6.3 (5.2–7.4)	4.8 (1.5–14.9)
CA 19-9 ≥35 kU/L IL-6 ≥5.6 ng/L	19	19	8.1 (2.3–13.9)	9.0 (3.0–26.9)

*IL-6* interleukin-6, *CA* carbohydrate antigen, *CI* confidence interval, *HR* hazard ratio, *OS* overall survival

## Discussion

The present results add new information about the two serum biomarkers CEA and CA 19-9, both of which are routinely used in the management of gastrointestinal cancers. The data provide evidence that elevated serum levels of CEA and CA 19-9, measured before the start of first-line chemotherapy, are independent, negative prognostic factors in patients with mCRC.

The relationship between elevated baseline CEA and impaired prognosis is in line with previous studies on patients with metastatic disease.^13,29,30^ The present results indicate that CEA is an independent prognostic biomarker in mCRC in adjusted models including *RAS/BRAF* mutation status. In addition, we want to bring attention to the favourable OS and the substantial proportion of long-term survivors in the patients with a baseline serum level of CEA below 5 µg/L.

The results showed that CA 19-9 can also give valuable prognostic information in mCRC. Elevated CA 19-9 serum levels were associated with impaired prognosis, consistent with reported data on mCRC in patients with unresectable liver metastases.^15,31^ This was independent of the CEA level at baseline and was confirmed in adjusted analyses. It has been suggested that CA 19-9 could be used to monitor the disease development in mCRC patients who have no elevation of CEA.^32^ Like CEA, low levels of CA 19-9 were associated with a quite high percentage of survivors beyond 5 years.

A major finding was that the present study suggests that elevated serum CA 19-9 can have a particular role in patients with *BRAF*-mutant mCRC. Thus, the median CA 19-9 level was numerically high in patients with *BRAF*-mutant tumours and 50% of those with an isolated elevated CA 19-9 (low CEA) had *BRAF*-mutant tumours. More importantly, an elevated CA 19-9 level was associated with short OS within the *BRAF*-mutant subgroup of patients. It is known that the presence of *BRAF* mutation in mCRC predicts impaired outcome;^2,4,6,33^ however, these cancers are heterogeneous.^9,19,20^ Although the underlying mechanisms are not clarified, the present findings suggest that CA 19-9 may help identify one subgroup of *BRAF*-mutant mCRC patients with particularly poor prognosis. On the other hand, the measured serum CA 19-9 did not predict initial response to chemotherapy, and an elevated level was not associated with resistance to chemotherapy, neither in patients with *BRAF*-mutant nor non-*BRAF*-mutant tumours.

We recently reported an association between high levels of inflammation biomarkers and poor prognosis in mCRC, and this was particularly prominent in the subset of patients with *BRAF*-mutant tumours.^22^ Thus, in mCRC patients with *BRAF*-mutant tumour, unlike *RAS*-mutant or *RAS*/*BRAF* double wild-type tumours, high serum IL-6 predicted markedly impaired survival. An obvious question, therefore, was whether high CA 19-9 identifies the same patients as elevated IL-6. The present data indicate that this is not the case and that IL-6 and CA 19-9 are independent biomarkers. Thus, patients with combined high CA 19-9 and high IL-6 had extremely short survival. Together, the results strengthen the indications that *BRAF*-mutant mCRC is heterogeneous and suggest that IL-6 and CA 19-9 are biomarkers of at least partly different mechanisms that underlie aggressive disease.

One of the findings in a recent large investigation analysing the outcomes of patients with mCRC with *BRAF* mutations was that the most marked divergence between the *BRAF*-mutant and wild-type cancers occurred following progression on or after benefit from first-line chemotherapy.^9^ Our results with serum CA 19-9, CEA, IL-6 and CRP strongly suggest that the prognostic prediction provided by these biomarkers also largely concerns advanced stages of mCRC. Thus, a marked difference in OS was noted between cases with high and low levels of the inflammatory markers despite relatively small differences in PFS.^22^ Furthermore, in the present study, focusing on OS, we observed that for both CEA and CA 19-9 the difference between the survival curves representing high and low levels of these markers was also reflected in long-term survival. This was so in the whole cohort and was quite dramatic when comparing low and high CA 19-9 in the subset of *BRAF*-mutant patients. Collectively, these data suggest that in addition to *BRAF* mutations, several other mechanisms, including inflammatory reactions as well as processes reflected in elevated levels of carbohydrate/glycoprotein markers, impair the outcome of mCRC by influencing the advanced course of the disease. These mechanisms are likely inherent in the phenotype of the particular cancer and some of them can potentiate the effect of mutated *BRAF*. Hopefully, better insights into these mechanisms may provide clues for developing novel therapeutic strategies for advanced mCRC.

We are aware of some limitations for CA 19-9 as a biomarker, since several benign diseases may also give rise to elevated levels, and CA 19-9 is not expressed in subjects with Le (a-b-) genotype in the Lewis blood group system. In this study, the Lewis blood-type status of the patient cohort was unknown. Nine percent of the patients in our cohort had no detectable CA 19-9 as judged by the lower reference value, and in a clinical routine setting, these patients will be reckoned as having a very low serum level of CA 19-9 below a detection limit, but it will not be possible to distinguish between patients with very low CA 19-9 levels from those who do not express CA 19-9 due to their Lewis blood type. This subgroup of patients had clinical characteristics and outcome that did not differ significantly from the total population with detectable levels of CA 19-9, suggesting that it included patients across the total population and not specifically those with a low CA 19-9 level.

Serum CA 19-9, alone or in combination with markers of systemic inflammation, can be useful in the management of patients with *BRAF*-mutant, primary unresectable mCRC. We believe that the findings are of general relevance, although the underlying chemotherapy regimen in the present study is not commonly used outside the Nordic countries. Elevated serum CA 19-9 level may help to identify patients with a highly aggressive disease, like those who should be considered for intensive first-line therapy, such as the triple combination FOLFOXIRI.^4,34^ In conclusion, integrated prognostic data, including the CEA and CA 19-9 biomarkers, may give more accurate information about the disease and can be useful in shared decision-making, enabling patient and clinician to establish an optimal treatment plan.

## Electronic supplementary material

Figure S1

Table S1

Table S2

Table S3

Table S4

Table S5

## Availability of data and material

The datasets generated during and/or analysed during the current study are not publicly available but are available from the corresponding author on reasonable request.
