# Supplementary figures and images for: Prognostic role of carcinoembryonic antigen and carbohydrate antigen 19-9 in metastatic colorectal cancer: a BRAF-mutant subset with high CA 19-9 level and poor outcome
Source: Br J Cancer. 2018 Jun 6;118(12):1609–16. doi: 10.1038/s41416-018-0115-9 (PMC6008450; doi:10.1038/s41416-018-0115-9)

**Figure S1.** Overall survival (OS) in 545 patients with serum samples analysed for CA 19-9.

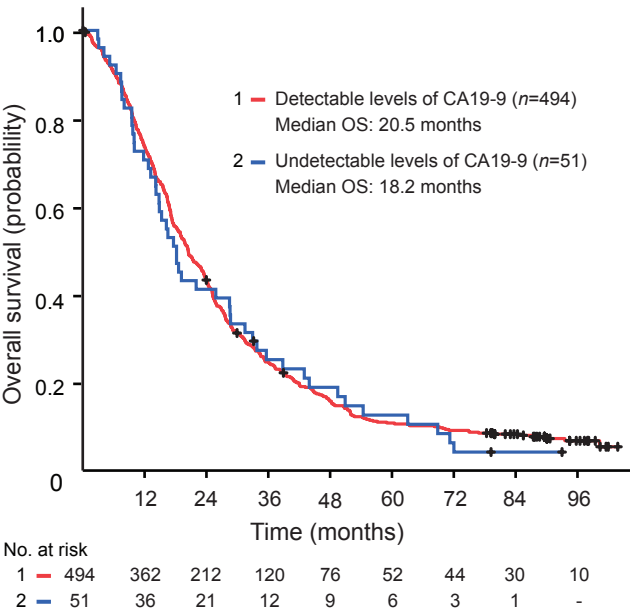

Supplement: Supplementary file 1 — Figure S1 [file 41416_2018_115_MOESM1_ESM.pdf]
