# Supplementary material for: Prognostic role of carcinoembryonic antigen and carbohydrate antigen 19-9 in metastatic colorectal cancer: a BRAF-mutant subset with high CA 19-9 level and poor outcome
Source: Br J Cancer. 2018 Jun 6;118(12):1609–16. doi: 10.1038/s41416-018-0115-9 (PMC6008450; doi:10.1038/s41416-018-0115-9)
Supplement: Supplementary file 2 — Table S1 [file 41416_2018_115_MOESM2_ESM.pdf]

Table S1. Serum level of CEA and CA 19-9 and clinical patient characteristics

Variables	Serum level of CEA (n=545)				Serum level of CA19-9 (n=494)			
	<5 µg/L		≥5 µg/L		<35 kU/L		≥35 kU/L	
	n	(%)	*n*	(%)	*n*	(%)	*n*	(%)
**WHO performance status**								
0	83	(23)	284	(77)	148	(45)	180	(55)
1	11	(7)	144	(93)	38	(26)	107	(74)
2	2	(9)	21	(91)	2	(10)	19	(90)
**Origin of primary tumour**								
Colon	59	(18)	262	(82)	105	(35)	192	(65)
Rectum	37	(17)	187	(84)	83	(42)	114	(58)
**Surgery of primary tumour**								
Resected	79	(21)	290	(79)	147	(44)	187	(56)
Not resected	17	(10)	159	(90)	41	(26)	119	(74)
**Time of metastases**								
Synchronous	60	(15)	329	(85)	109	(31)	238	(69)
Metachronous	36	(23)	120	(77)	79	(54)	68	(46)
**Type of metastases**								
Liver-only	14	(14)	88	(86)	33	(37)	56	(63)
Lung-only	8	(33)	16	(67)	15	(71)	6	(29)
Other-sites-only	6	(21)	23	(79)	12	(44)	15	(56)
Multiple sites	68	(17)	322	(83)	128	(36)	229	(64)
**Alkaline phosphatase level**								
Normal	76	(27)	210	(73)	132	(51)	126	(49)
> UNL	20	(8)	239	(92)	56	(24)	180	(76)
**Platelet count**								
≤400/nL	77	(20)	306	(80)	142	(41)	205	(59)
>400/nL	19	(12)	143	(88)	46	(31)	101	(69)
**White blood cell count**								
≤10/nL	87	(21)	326	(79)	161	(44)	208	(56)
>10/nL	9	(7)	123	(93)	27	(22)	98	(78)
***RAS/BRAF* mutation status**								
RAS/BRAF wild-type	27[*]	(15)	159[*]	(85)	72[‡]	(42)	99[‡]	(58)
RAS mutation	40[*]	(20)	161[*]	(80)	65[‡]	(36)	114[‡]	(64)
BRAF mutation	18[*]	(34)	35[*]	(66)	18[‡]	(37)	31[‡]	(63)
**CRP level**								
≤10 mg/L	55[†]	(28)	139[†]	(72)	91[§]	(52)	85[§]	(48)
>10 mg/L	27[†]	(12)	207[†]	(88)	63[§]	(30)	149[§]	(70)

Abbreviations: CRP, C-reactive protein; UNL, Upper normal limit; WHO, World Health Organization.
[*]Analysed for *RAS/BRAF* mutations (*n*=440), [†]CRP (*n*=428), [‡]*RAS/BRAF* mutations (*n*=399), [§]CRP (*n*=388).
