# Supplementary material for: Prognostic role of carcinoembryonic antigen and carbohydrate antigen 19-9 in metastatic colorectal cancer: a BRAF-mutant subset with high CA 19-9 level and poor outcome
Source: Br J Cancer. 2018 Jun 6;118(12):1609–16. doi: 10.1038/s41416-018-0115-9 (PMC6008450; doi:10.1038/s41416-018-0115-9)
Supplement: Supplementary file 3 — Table S2 [file 41416_2018_115_MOESM3_ESM.pdf]

Table S2. Association between CEA and overall survival. Adjusted models								
Variables	Study populations							
	CEA/CA 19-9-analysed (*n*=545)				CEA/CRP- and *RAS*/*BRAF*-analysed (*n*=428)			
	n	**HR**	**95% CI**	***P***	***n***	**HR**	**95% CI**	***P***
**Serum level of CEA**								
<5 µg/L	96	1			82	1		<0.001
≥5 µg/L	449	1.47	1.13-1.90	0.004	346	1.78	1.34-2.37	
**WHO performance status**								
0	367	1			291	1		
1	155	1.51	1.24-1.85	<0.001	118	1.46	1.47-1.85	0.002
2	23	2.60	1.68-4.01	<0.001	19	2.54		<0.001
**Alkaline phosphatase level**								
Normal	286	1			233	1		<0.001
> UNL	259	1.68	1.40-2.03	<0.001	195	1.58	1.26-1.99	
***RAS*/*BRAF* mutation status**								
RAS/*BRAF* wild-type					182	1		
RAS mutant					196	1.38	1.11-1.71	0.003
BRAF mutant					50	3.78	2.70-5.30	<0.001
**CRP level**								
CRP per category*					428	1.16	1.04-1.29	0.008

Abbreviations: CRP, C-reactive protein; CI, Confidence interval; HR, Hazard ratio; OS, Overall survival; UNL, Upper normal limit; WHO, World Health Organization. *CRP categories: [0 to 10 mg/L], [from 10 to 30 mg/L], [from 30 to 60 mg/L], [from 60 mg/L and higher].
