# Supplementary material for: Prognostic role of carcinoembryonic antigen and carbohydrate antigen 19-9 in metastatic colorectal cancer: a BRAF-mutant subset with high CA 19-9 level and poor outcome
Source: Br J Cancer. 2018 Jun 6;118(12):1609–16. doi: 10.1038/s41416-018-0115-9 (PMC6008450; doi:10.1038/s41416-018-0115-9)
Supplement: Supplementary file 4 — Table S3 [file 41416_2018_115_MOESM4_ESM.pdf]

Table S3. Serum level of CA 19-9 and treatment outcome in subgroups of patients based on tumour *RAS/BRAF* mutation status															
**Treatment outcome**	**Patient populations**														
	**CA 19-9 analysed and detectable**			***RAS/BRAF* analysed**			***RAS/BRAF* wild-type**			***RAS* Mutant**			***BRAF* mutant**		
	n	**Outcome *n* (%)**		***n***	**Outcome *n* (%)**		***n***	**Outcome *n* (%)**		***n***	**Outcome *n* (%)**		***n***	**Outcome *n* (%)**	
**Confirmed response (CR+ PR)**															
All	494	234	(47)	399	191	(48)	171	102	(60)	179	78	(44)	49	11	(22)
CA19-9 <35 kU/L	188	93	(50)	155	78	(50)	72	46	(64)	65	28	(43)	18	4	(22)
CA19-9 ≥35 kU/L	306	141	(46)	244	113	(46)	99	56	(57)	114	50	(44)	31	7	(23)
**Disease control (CR+PR+SD)**															
All	494	428	(87)	399	345	(87)	171	153	(90)	179	155	(87)	49	37	(76)
CA19-9 <35 kU/L	188	168	(89)	155	137	(88)	72	66	(92)	65	56	(86)	18	15	(83)
CA19-9 ≥35 kU/L	306	260	(85)	244	208	(85)	99	87	(88)	114	99	(87)	31	22	(71)
**Direct progression**															
All	494	26	(5)	399	21	(5)	171	7	(4)	179	8	(5)	49	6	(12)
CA19-9 <35 kU/L	188	7	(4)	155	7	(5)	72	2	(3)	65	4	(6)	18	1	(6)
CA19-9 ≥35 kU/L	306	19	(6)	244	14	(6)	99	5	(5)	114	4	(4)	31	5	(16)
**Secondary resection of metastases**															
All	494	41	(8)	399	35	(9)	171	23	(14)	179	12	(7)	49	0	
CA19-9 <35 kU/L	188	18	(10)	155	16	(10)	72	12	(17)	65	4	(6)	18		
CA19-9 ≥35 kU/L	306	23	(8)	244	19	(8)	99	11	(11)	114	8	(7)	31		

Abbreviations: CR, Complete response; PR, Partial response; SD, Stable disease.
