# Supplementary material for: Prognostic role of carcinoembryonic antigen and carbohydrate antigen 19-9 in metastatic colorectal cancer: a BRAF-mutant subset with high CA 19-9 level and poor outcome
Source: Br J Cancer. 2018 Jun 6;118(12):1609–16. doi: 10.1038/s41416-018-0115-9 (PMC6008450; doi:10.1038/s41416-018-0115-9)
Supplement: Supplementary file 5 — Table S4 [file 41416_2018_115_MOESM5_ESM.pdf]

Table S4. Serum level of CEA and treatment outcome in subgroups of patients based on tumour *RAS/BRAF* mutation status										
**Treatment outcome**	**Patient populations**									
	**CEA analysed**		***RAS/BRAF* analysed**		***RAS/BRAF* wild-type**		***RAS* Mutant**		***BRAF* mutant**	
	n	**Outcome *n* (%)**	***n***	**Outcome *n* (%)**	***n***	**Outcome *n* (%)**	***n***	**Outcome *n* (%)**	***n***	**Outcome *n* (%)**
**Confirmed response (CR+ PR)**										
All	545	252 (46)	440	205 (47)	186	108 (58)	201	86 (43)	53	11 (21)
CEA <5 mg/L	96	50 (52)	85	44 (52)	27	18 (67)	40	21 (53)	18	5 (28)
CEA ≥5 mg/L	449	202 (45)	355	161 (45)	159	90 (57)	161	65 (40)	35	6 (17)
**Disease control (CR+PR+SD)**										
All	545	390 (87)	440	380 (86)	186	165 (89)	201	175 (87)	53	40 (76)
CEA <5 mg/L	96	83 (87)	85	75 (88)	27	24 (89)	40	37 (93)	35	26 (74)
CEA ≥5 mg/L	449	390 (87)	355	305 (86)	159	141 (89)	161	138 (86)	18	14 (78)
**Direct progression**										
All	545	30 (6)	440	25 (6)	186	10 (5)	201	9 (5)	53	6 (11)
CEA <5 mg/L	96	6 (6)	85	6 (7)	27	2 (7)	40	2 (5)	35	2 (11)
CEA ≥5 mg/L	449	24 (5)	355	19 (5)	159	8 (5)	161	7 (4)	18	4 (11)
**Secondary resection of metastases**										
All	545	45 (8)	440	38 (9)	186	24 (13)	201	13 (7)	53	1 (2)
CEA <5 mg/L	96	9 (9)	85	9 (11)	27	6 (22)	40	3 (8)	35	0
CEA ≥5 mg/L	449	36 (8)	355	29 (8)	159	18 (11)	161	10 (6)	18	1 (3)

Abbreviations: CR, Complete response; PR, Partial response; SD, Stable disease.
