# Supplementary material for: Prognostic role of carcinoembryonic antigen and carbohydrate antigen 19-9 in metastatic colorectal cancer: a BRAF-mutant subset with high CA 19-9 level and poor outcome
Source: Br J Cancer. 2018 Jun 6;118(12):1609–16. doi: 10.1038/s41416-018-0115-9 (PMC6008450; doi:10.1038/s41416-018-0115-9)
Supplement: Supplementary file 6 — Table S5 [file 41416_2018_115_MOESM6_ESM.pdf]

Table S5. Relationship between best response and changes in serum level of CEA and CA 19-9 before start of treatment to cycle 4 (8 weeks of treatment) in patients with elevated CEA (≥ 5µg/L) or CA 19-9 (≥ 35 kU/L) at baseline

Patients with baseline CEA ≥ 5 µg/L (*n*=384)			Change in serum level of CEA				Patients with baseline CA 19-9 ≥ 35 kU/L (*n*=260)			Change in serum level of CA 19-9			
			Drop		Rise					Drop		Rise	
**Clinical response**	***n***	**(%)**	***n***	**(%)**	***n***	**(%)**	**Clinical response**	***n***	**(%)**	***n***	**(%)**	***n***	**(%)**
**CR+PR**	187	(49)	177	(95)	10	(5)	**CR+PR**	130	(50)	124	(95)	6	(5)
**SD**	170	(44)	128	(75)	42	(25)	**SD**	110	(42)	87	(79)	23	(21)
**PD**	20	(5)	6	(30)	14	(70)	**PD**	16	(6)	8	(50)	8	(50)
**NE**	7	(2)					**NE**	4	(2)				

Abbreviations: CR, Complete response; NE, Not evaluable; PD, Progressive disease; PR, Partial response; SD, Stable disease.
